# Identification key to Nephtyidae (Annelida) of the Sea of Okhotsk

**DOI:** 10.3897/zookeys.684.12180

**Published:** 2017-07-11

**Authors:** Inna L. Alalykina, Nataliya Yu. Dnestrovskaya, Igor A. Jirkov

**Affiliations:** 1 A.V. Zhirmunsky Institute of Marine Biology, National Scientific Center of Marine Biology, Far Eastern Branch, Russian Academy of Sciences, ul. Pal’chevskogo 17, Vladivostok, 690041 Russia; 2 Faculty of Biology, Lomonosov Moscow State University, Moscow, 119234 Russia

**Keywords:** *Aglaophamus*, *Micronephthys*, *Nephtys*, Polychaeta, north-western Pacific

## Abstract

Currently, 15 species of Nephtyidae (Annelida) are known from the Sea of Okhotsk (north-western Pacific). A new user-friendly identification key is presented with a brief description for each species. The taxonomic positions of three closely related species, *Nephtys brachycephala* Moore, 1903, *N. schmitti* Hartman, 1938 and *N. paradoxa* Malm, 1874, are revised. The distributions of two species, *Nephtys discors* Ehlers, 1968 and *N. assignis* Hartman, 1950, are discussed.

## Introduction

Nephtyids are benthic polychaetes occurring worldwide from the intertidal to abyssal depths and mainly inhabiting soft sediments. Most of them are actively burrowing carnivores, although several species may be subsurface deposit feeders (Jumars et al. 2015). The smallest species are less than 10 mm long, while others can be rather large: the largest species from the Sea of Okhotsk may grow up to 300 mm long.

The family Nephtyidae includes approximately 140 species (Read and Fauchald 2017). More than 90 species occur in Pacific waters (Hartman 1938, 1950, Hilbig 1997, Murray et al. 2015). The first species of this family known from the Sea of Okhotsk was *Nephtys ciliata* (O.F. Müller, 1789), collected by Moore (1903) off the west coast of Kamchatka onboard the R/V “Albatross”. Later, Uschakov (1950, 1953, 1955), Imajima (1961) and Buzhinskaja (1985) added several more species to the local nephtyid fauna.

At present, 15 species of Nephtyidae are known from the Sea of Okhotsk. Thirteen of them belong to the most diverse genus *Nephtys*: *N. assignis* Hartman, 1950, *N. brachycephala* Moore, 1903, *N. caeca* (Fabricius, 1780), *N. californiensis* Hartman, 1938, *N. ciliata*, *N. longosetosa* Örsted, 1842, *N. neopolybranchia* Imajima and Takeda, 1987, *N. paradoxa* Malm, 1874, *N. pente* Rainer, 1984, *N. punctata* Hartman, 1938, *N. rickettsi* Hartman, 1938, *N. sachalinensis* Alalykina and Dnestrovskaya, 2015 and *N. schmitti* Hartman, 1938; one species belongs to *Aglaophamus*: *A. malmgreni* (Théel, 1879); and one to *Micronephthys*: *M. minuta* (Théel, 1879) (Buzhinskaja 2013, Alalykina and Dnestrovskaya 2015).

Herein, an illustrated key is provided to identify species known from the Sea of Okhotsk. This key is based mainly on external morphological characters. In brief species descriptions characters of the pharynx are also included, which are easily visible by dissection and highlighted by staining. This review facilitates the creation of a valid checklist of Nephtyidae species for this region.

## Remarks on the key

Nephtyids are rather similar in their gross morphology and often difficult to distinguish. The main taxonomic characters are the position of the first branchiae, their shape and the number of branchiferous chaetigers, parapodial features (shape and size of acicular lobes, pre- and postacicular lobes, characters of chaetae) and pharynx structure.

**Figure 1.**
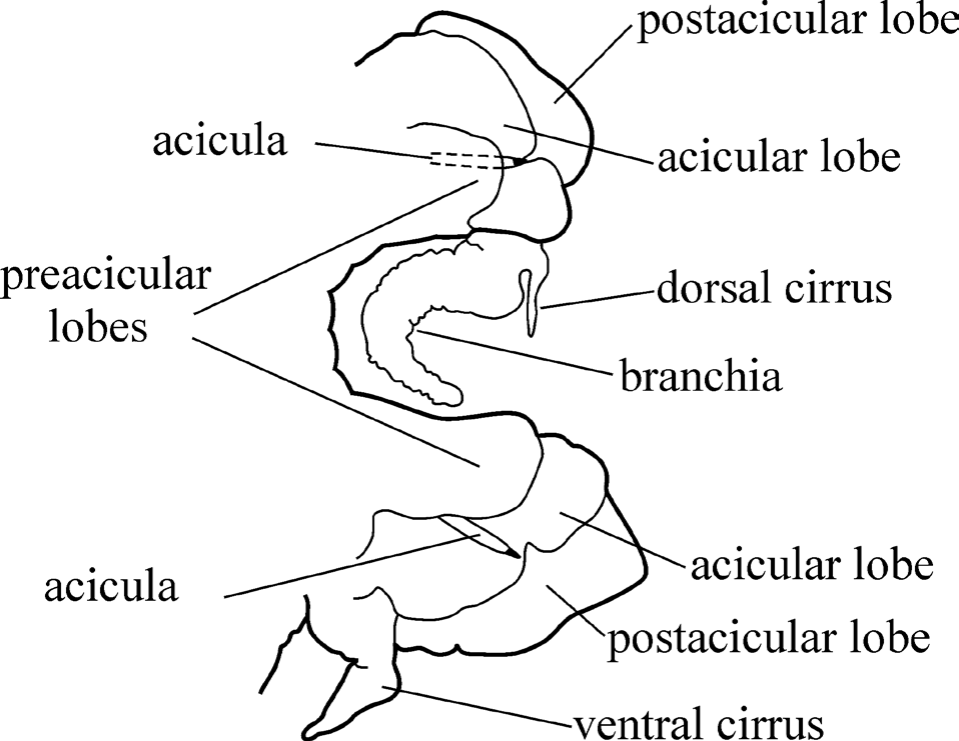
Explanation of main parapodial terminology used.

**Figure 2.**
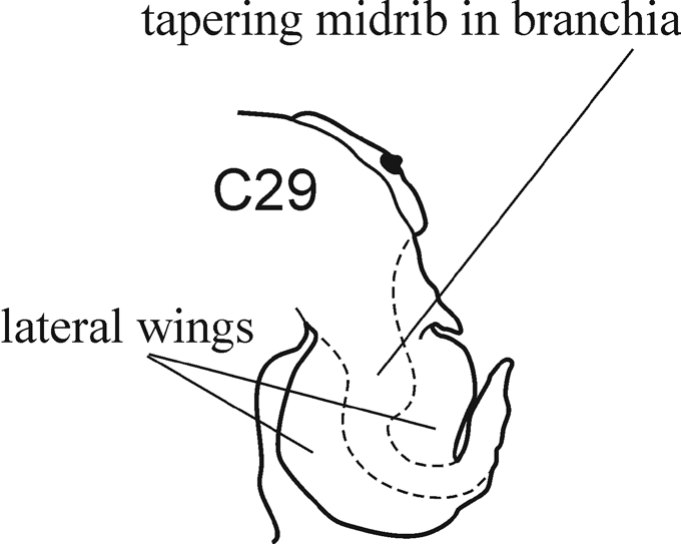
Shape of foliaceous branchia with midrib and wings.

The parapodia are biramous. Both noto- and neuropodia consist of acicular, pre- and postacicular lobes, and dorsal (notopodial) and ventral (neuropodial) cirri. The acicular lobes are supported by one acicula and may be conical, rounded or bilobed (Fig. 1). The branchiae (also called interramal cirri), are inserted below the dorsal cirri; they may be involute or recurved, slender and digitiform, or basally inflated and foliaceous. Foliaceous branchiae may be evenly flattened or with a thick tapering midrib and thin lateral wings (Fig. 2). A small spherical papilla may be present at the base of a branchia under the notopodial cirrus. The shape and proportions of these structures vary along the body, so they should be examined on the chaetigers that are recommended in the key.

**Figure 3.**
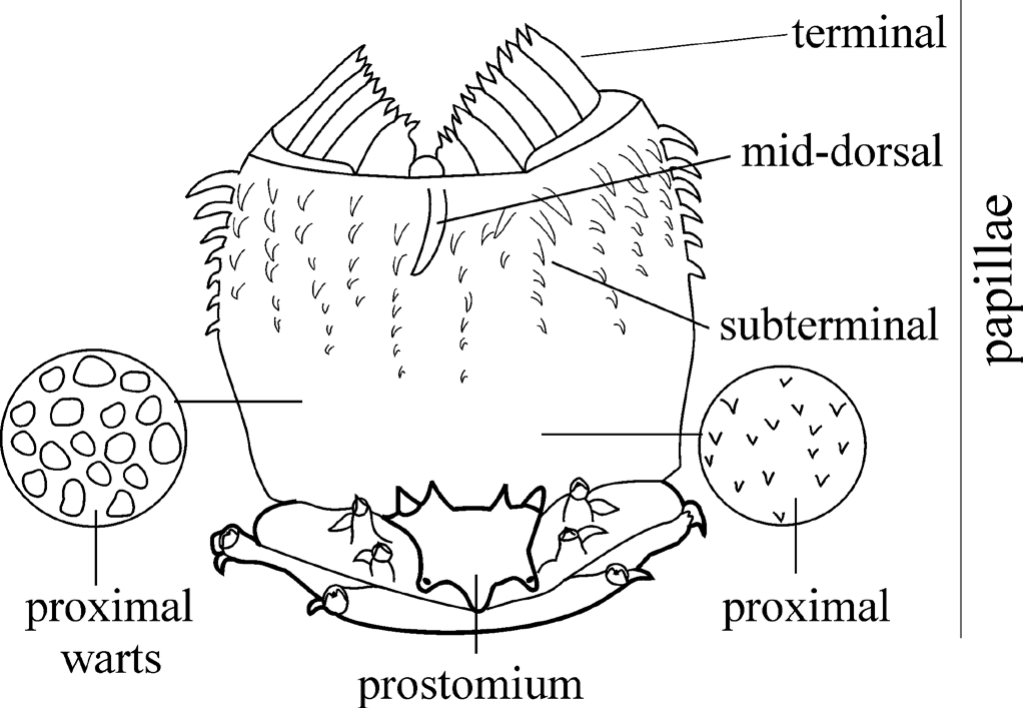
Explanation of main terminology of pharynx used.

The pharynx is a large eversible muscular proboscis, covered with soft papillae located in different areas that can be seen when everted (Fig. 3) and usually with one pair of small subterminal jaws located inside (visible with dissection). The anterior margin is surrounded by 18–20 bifid terminal papillae separated dorsally and ventrally by gaps; each gap may bear a single conical papilla. The subterminal region has 14 to 22 longitudinal rows of conical to digitiform papillae decreasing in size towards the base of the pharynx (absent in *Inermonephtys*). A single longer subterminal papilla may be present mid-dorsally and mid-ventrally. The proximal surface may be smooth or covered with small warts (flat outgrowths) or small papillae (conical or rounded) which slightly rise above the surface.

**Figure 4.**
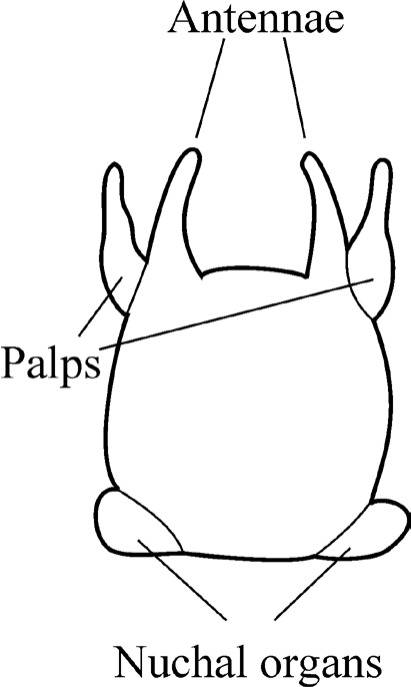
Explanation of prostomium terminology used.

The prostomium is subquadrangular to subpentagonal (shape depends on whether the proboscis is everted or not). A pair of conical antennae is present in the anterior corners of the prostomium (absent in *Inermonephtys*). A pair of palps is inserted ventrolaterally (may be bifid in *Micronephthys*). A pair of nuchal organs is located dorsolaterally on the posterior margin of the prostomium (Fig. 4). Pigment spots on prostomium (if present) may fade. It is strongly recommended to examine several specimens, rather than a single individual for identification. Staining with methylene blue makes morphological characters more visible. The segment on which the branchiae begin should be checked on both sides of worm. Several undamaged parapodia from both sides of the worm should be examined.

The morphological details of the parapodia can usually be seen under the stereomicroscope without preparing slides. All parapodia are shown in anterior view. Pharynx dissection is not always necessary but may be useful to confirm identifications. It is important to mention that not all characters are developed in juveniles, and it is not always possible to identify fragmented animals without specialized training.

Each species of *Nephtys* is here provided with a brief description and distribution. All figures are original except for that of *N. brachycephala* (after Uschakov 1950) and *N. assignis* (after Hartman 1950). Abbreviations: AMNH – American Museum of Natural History, New York, USA; USNM – National Museum of Natural History, Washington, DC, USA; MCZ – Museum of Comparative Zoology of Harvard University, Cambridge, MA, USA; C – chaetiger. Abbreviations with numbers denote the chaetiger, i.e. C3 means the third chaetiger. All features used in the couplets are shown in the figures nearby.

No key is complete and perfect. The key given below should be used with caution and collated with descriptions of the species concerned. If you have any difficulties, do not hesitate to contact us by e-mail or by other means.

## Taxonomic remarks

1. *Nephtys brachycephala* Moore, 1903 was originally described from Sagami Bay and recorded only a few times subsequently from the northern part of the Sea of Japan (Annenkova 1937, 1938), the Sea of Okhotsk (Uschakov 1950, 1955), the Bering Sea (Levenstein 1961), the Pacific coast of Japan (Imajima and Hartman 1964) and British Columbia (Berkeley 1966). It was questionably referred to *N. paradoxa* Malm, 1874 by Pettibone (1954). Recently, Ravara et al. (2010) also synonymized *N. brachycephala* sensu Uschakov with *N. paradoxa*, based on the literature, without examination of Uschakov’s specimens.

*Nephtys schmitti* Hartman, 1938 from Alaska was also synonymized with *N. paradoxa* by Imajima and Takeda (1987) tentatively as they had not examined the type material. Hilbig (1997) examined the holotype of *N. schmitti* and specimens of *N. paradoxa* from Alaska and concluded that they represented two valid species. Later, Ravara et al. (2010) examined the Alaskan specimens of *N. schmitti* (including the holotype) and specimens of *N. paradoxa* from Europe (including the type locality) and found no significant differences between these specimens. However they considered *N. schmitti* to belong to a *N. paradoxa* species complex that seemed to have a worldwide distribution, and that the taxonomic status of this species complex should be carefully revised with examination of more specimens.

All three closely related species *N. brachycephala*, *N. schmitti* and *N. paradoxa*, have foliaceous branchiae and similar parapodial features. However, in contrast to *N. paradoxa*, the two other species (*N. brachycephala* and *N. schmitti*) have leaf-like branchiae with a thick tapering midrib that runs through its centre. Our examination of material from the Arctic, North Atlantic and north-western Pacific (497 specimens) has indicated that the foliaceous branchiae of *N. paradoxa* specimens lack the tapering midrib.

Furthermore, we examined the type material of both *N. brachycephala* (USNM 15722) and *N. schmitti* (USNM 20323) and found considerable differences between these species. *Nephtys schmitti* has foliaceous branchiae between chaetigers 12–35. From chaetiger 36 the foliaceous lobes suddenly disappear and the large cylindrical branchiae decrease gradually in size posteriorly, absent in the last 8–9 chaetigers. In *N. brachycephala*, the foliaceous branchiae start at chaetiger 15 and decrease gradually in size to chaetiger 50 and only the shortened midribs remain until chaetiger 55–58; branchiae are absent in posterior segments. We consider all these to be separate species.

2. *Nephtys discors* Ehlers, 1868 was originally described from Maine, USA and its distribution appears to be restricted to the north-western Atlantic. Specimens of *N. discors* from the west coast of Kamchatka (Sea of Okhotsk) (Imajima 1961) were examined and synonymized with *N. assignis* Hartman, 1950 by Banse (1972). We examined the type material of *N. discors* (MCZ IZ 700 and MCZ IZ 91707) and came to the same conclusion as Banse (1972). *Nephtys discors* sensu Imajima and Takeda (1987) from the east coast of Hokkaido (off the Notsuke Peninsula, on the southern edge of the Sea of Okhotsk, north-western Pacific) appeared to belong to the same species as the one from the west coast of Kamchatka, therefore was also considered as *N. assignis* (Ravara, 2010). Thus we excluded *N. discors* from our key and included *N. assignis*.

Nevertheless, both species remain valid. *Nephtys assignis* is a Pacific species with the initially minute branchiae increasing in size through segments 12–20; it has a proximally smooth pharynx and posterior parapodia with well-developed branchiae. *Nephtys discors* is a West Atlantic species with the branchiae best developed on the anterior third of the body and rudimentary in the posterior half; the pharynx is covered with proximal warts.

## Key to nephtyid species from the Sea of Okhotsk

**Table:** 

1	3–9 branchiferous chaetigers in worms longer than 3 mm; no more than 34 chaetigers. Up to 16 mm long (usually shorter)	***Micronephthys minuta***
(Key figure 5) Small worms with body length up to 16 mm (Théel 1879), up to 34 chaetigers. Branchiae from C6–C9 to C10–C14 (Dnestrovskaya and Jirkov 2001) small, wrinkled, always shorter than notopodia. Palps bifid (arrow), upper branch twice as long as lower, visible in well preserved worms only). Parapodial preacicular and postacicular lobes rudimentary; acicular lobes conical. Pharynx with elongate mid-dorsal papilla; proximal region smooth. Arcto-boreal, shelf.		
–	Usually several tens of chaetigers with branchiae; up to 100 chaetigers or more and may be over 200 mm long in adults (in juveniles number of chaetigers may be low, but just before the pygidium there is a growing zone with numerous developing chaetigers)	**2**
2(1)	Branchiae of median parapodia curved inward	***Aglaophamus malmgreni***
(Key figure 6) Body length up to 195 mm, up to 87 chaetigers. Branchiae from C9–C22, to C22–C47, always longer than notopodia. All preacicular and postacicular lobes well developed but always lower than acicular lobes. Neuropodial preacicular lobes in anteriormost chaetigers (before branchiae) bilobed with small lower and larger upper parts; in median branchiferous chaetigers rounded; and in posteriormost chaetigers rudimentary. Notopodial postacicular lobes bilobed with equal parts in anterior and median parapodia, and with small lower and larger upper parts in posterior chaetigers. Acicular lobes conical. Proximal region of pharynx smooth; elongate mid-dorsal and mid-ventral subterminal papillae absent. Arcto-boreal, lower shelf, slope and deeper.		
–	(Key figure 7) Branchiae of median parapodia curved outward	***Nephtys***...**3**
3(2)	(Key figure 8) Neuropodial postacicular lobes of median chaetigers (after C30) almost equal or shorter than acicular lobes	**4**
–	(Key figure 9) Neuropodial postacicular lobes of median chaetigers (after C30) distinctly longer than acicular lobes	**10**
4(3)	Branchiae from C3 or C4	**5**
–	Branchiae from C5 or posteriorly	**6**
5(5)	In median parapodia, acicular lobes bilobed; dorsal and ventral parapodial cirri of C1 (arrow) long; subsequent chaetigers with short dorsal and ventral cirri; in median and posterior chaetigers cirri long again	***N. californiensis***
(Key figure 10) Large worms with body length up to 300 mm, up to 160 chaetigers (Hilbig 1997). Branchiae from C3 or C4 to posterior end of body, longer than notopodia in median region. Large worms with small spherical papilla at base of branchia under notopodial cirrus (arrow). Prostomium always with small dark spot in central part and sometimes with spread-eagle pigmentation pattern in posterior part (all fading during prolonged storage). Parapodial preacicular lobes low; dorsal part of each neuropodial preacicular lobe collar like, surrounding corresponding acicular lobe. Neuropodial postacicular lobes somewhat longer than notopodial postacicular lobes; after C30 both subequal in length or slightly longer than acicular lobes. Ventral cirri of median and posterior chaetigers slender and digitate, somewhat larger than corresponding dorsal cirri. Postacicular chaetae numerous, extremely long, soft and flexible. Proximal region of pharynx smooth; elongate mid-dorsal and mid-ventral subterminal papillae absent. Subtropical-boreal, intertidal to shelf.		
–	Acicular lobes rounded-conical or conical throughout; dorsal and ventral parapodial cirri short throughout	***N. neopolybranchia***
(Key figure 11) Small worms with body length up to 24 mm, up to 62 chaetigers. Branchiae small (always shorter than notopodia), from C3 to near posterior end. Prostomium with small dark spot or cross in central part (fading during prolonged storage). Parapodial preacicular lobes rudimentary. Anterior neuropodial postacicular lobes (before C12) distinctly longer than acicular lobes, posteriorly subequal in length, or slightly shorter than acicular lobes. Proximal region of pharynx with minute warts; elongate mid-dorsal and mid-ventral subterminal pharyngeal papillae absent. Boreal and subtropical, intertidal to upper shelf.		
6(4)	(Key figure 12) Branchiae initially small, shorter than dorsal cirri, gradually increasing in size; in median chaetigers more or less foliaceous	**7**
–	(Key figure 13) Branchiae of anterior chaetigers longer than dorsal cirri, cirriform throughout	**9**
7(6)	(Key figure 14) Foliaceous branchiae with a thick tapering midrib and thin lateral wings	**8**
–	Foliaceous branchiae without a tapering midrib and lateral wings, their thickness not varying from the centre to the edges	***N. paradoxa***
(Key figure 15) Body length up to 200 mm, up to 150 chaetigers (Rainer 1991). Branchiae from C7–C12, minute at first, gradually increasing in size to C25–C27, in median chaetigers often (but not always) more or less foliaceous, rounded fleshy, without tapering midrib or thin lateral wings. Parapodial preacicular lobes rudimentary. Anterior notopodial acicular lobes sometimes slightly bilobed, posteriorly always rounded-conical. Postacicular lobes of anterior and median parapodia subequal in length to or slightly longer than acicular lobes, posteriorly shorter than acicular lobes. Pharynx with short mid-dorsal subterminal papilla; in large worms proximal region of pharynx sometimes covered with small conical papillae. Arcto-boreal lower shelf.		
8(7)	Branchiae from C7 or C8; thin lateral wings of well-developed branchiae reaching to approx. half way along midrib or slightly higher, absent exactly from C36	***N. schmitti***
(Key figure 16) Body length up to 90 mm, approx. 100 chaetigers (Hilbig 1997). Branchiae minute at first, increasing in size gradually; well-developed branchiae with thick tapering midrib and thin lateral wings reaching half way along midrib from C10–C15 to C35. Exactly from C36 branchiae without lateral wings, long and digitiform, posteriorly decreasing in size gradually, absent from last 8–9 chaetigers. Parapodial preacicular lobes low throughout, poorly developed. Acicular lobes of anterior parapodia rounded (notopodial acicular lobes sometimes slightly bilobed), rounded-conical in median region and conical in posterior chaetigers. Anterior parapodial postacicular lobes (before C30) subequal in length, or slightly longer than, acicular lobes, posteriorly shorter than acicular lobes. Both dorsal and ventral cirri of anterior and median chaetigers short, broadly conical, tapering to pointed tip, posteriorly decreasing in size to small and conical. Pharynx proximal region wrinkled, without papillae; elongate mid-dorsal subterminal papilla absent. Boreal slope and upper bathyal.		
–	Branchiae from C4 or C5; thin lateral wings of well-developed branchiae reach almost to the distal end of the midrib except for a slightly projecting tip	***N. brachycephala***
(Key figure 17) Body length more than 64 mm, more than 60 chaetigers (Moore 1903). Branchiae minute at first, increasing in size gradually; well-developed branchiae (after C15) broadly foliaceous with thick tapering midrib and thin lateral wings reaching almost to distal end of midrib except for slightly projecting tip. Posteriorly branchiae decreasing in size very gradually: posterior to C41, branchiae rounded fleshy, without wings; from C51, only small cylindrical midrib present. Branchiae absent after C55–C58. Parapodial rami widely separated, noto- and neuropodia subequal in size. Pre- and postacicular lobes poorly developed, subequal in length to or shorter than acicular lobes. Acicular lobes of anterior parapodia rounded (in notopodia sometimes slightly bilobed), rounded-conical in median region and conical in posterior chaetigers. Elongate mid-dorsal subterminal papilla absent. Subtropical-boreal shelf.		
9(6)	Branchiae from C5 or C6, they continue to C75–C85 as structures longer than dorsal cirri; dorsal cirri of median chaetigers short, broadly triangular	***N. pente***
(Key figure 18) Body length up to 140 mm, up to 90 chaetigers (Rainer 1991). Branchiae decreasing in size to minute knob (shorter than dorsal cirri) after C75–C85 and then completely absent. Parapodial preacicular lobes low throughout, poorly developed. In anterior and median chaetigers acicular lobes deeply bilobed, posteriorly indentation of acicular lobes becoming shallower, but may be visible up to last chaetigers. Postacicular lobes rounded, in anterior and median parapodia slightly longer or subequal in length to acicular lobes, in posterior chaetigers equal in length to or shorter than acicular lobes. Pharynx with long mid-dorsal subterminal papilla, proximal region in adults with flattened distally rounded papillae (conical in juveniles). Arcto-boreal upper shelf.		
–	Branchiae from C8–C12 (rarely from C7), they continue as structures longer than dorsal cirri to C45–C55; dorsal cirri of median chaetigers long and cirriform	***N. ciliata***
(Key figure 19) Body length up to 170 mm, up to 94 chaetigers. After C45–C55 branchiae decreasing in size to small knob (shorter than dorsal cirrus), and then completely absent. Notopodial preacicular lobes rudimentary, neuropodial preacicular lobes low, but distinct. Acicular lobes bilobed in anterior and median region, rounded in posterior chaetigers. Notopodial postacicular lobes shorter or subequal in length to acicular lobes, neuropodial postacicular lobes subequal in length to or slightly longer than acicular lobes. Pharynx with long mid-dorsal subterminal papilla, proximal region covered with small conical papillae. Arcto-boreal lower shelf.		
10(3)	Neuropodial postacicular lobes of median chaetigers with distinct indentation on the ventral side (arrow); branchiae from C3 to near posterior end (rarely from C4 – usually in small worms)	***N. longosetosa***
(Key figure 20) Body length up to 174 mm, up to 121 chaetigers. Parapodial preacicular lobes low and rounded, in large worms sometimes slightly bilobed in notopodia. Acicular lobes of anterior chaetigers (and in median chaetigers in large worms) bilobed. Neuropodial postacicular lobes of median and posterior chaetigers much longer than acicular and notopodial lobes, with rounded tips and distinct indentation on ventral side (around C40). Pharynx with long mid-dorsal subterminal papilla, proximal region smooth or covered with flat warts in large specimens. Boreal shelf.		
–	(Key figure 21) No indentation on ventral side of neuropodial postacicular lobes; branchiae from C4 or later	**11**
11(10)	(Key figure 22) Interramal (internal) parts of acicular lobes enlarged (arrows), distinctly higher than dorsal and ventral (outer) parts of acicular lobes	**12**
–	(Key figure 23) Interramal parts of acicular lobes not enlarged, subequal in size and shape to dorsal and ventral (outer) lobes	**13**
12(11)	Branchiae from C6; rounded interramal parts of acicular lobes enlarged in anterior and median chaetigers	***N. rickettsi***
(Key figure 24) Body length up to 300 mm, up to 120 chaetigers (Hartman 1938), according to our data up to 127 chaetigers. Branchiae of median chaetigers basally thickened, fleshy with narrow tips, shorter and flattened towards tail; in last 15–17 chaetigers shorter than dorsal cirri. In large animals, dorsal cirri of first few (five or more) chaetigers subglobular, triangular-foliaceous towards tail; conical in all chaetigers of small worms. Parapodial preacicular lobes rudimentary. Neuropodial acicular lobes bilobed in anterior chaetigers, notopodial acicular lobes also bilobed in median chaetigers, obliquely-oval towards tail. Postacicular lobes much longer than acicular lobes, rounded-foliaceous. Pharynx without mid-dorsal subterminal papilla; median part of pharynx with small conical papillae, proximal region smooth. Arcto-boreal, shelf.		
–	Branchiae from C4; rounded interramal parts of acicular lobes greatly enlarged up to posterior end	***N. sachalinensis***
(Key figure 25) Body length up to 107 mm, up to 112 chaetigers (Alalykina and Dnestrovskaya 2015). Dorsal cirri triangular-foliaceous in anterior chaetigers, elongate and subulate in median and posterior chaetigers. Parapodial preacicular lobes simple, low, rounded. Acicular lobes bilobed from C3–C4 to near posterior end. In median chaetigers notopodial postacicular lobes obliquely rounded, slightly longer or equal to acicular lobes. Neuropodial postacicular lobes elongated-triangular with obtuse tips, almost twice as long as acicular lobes. Pharynx without mid-dorsal subterminal pharyngeal papilla; proximal and median regions of pharynx with small rounded papillae. Boreal, upper sublittoral.		
13(11)	(Key figure 28) Branchiae from C7–C9; neuropodial postacicular lobes of median chaetigers distinctly longer than notopodial	***N. punctata***
(Key figure 26) Body length up to 100 mm, up to 108 chaetigers (Hilbig 1997). Branchiae only slightly larger than dorsal cirri at first, best developed from around C20, continuing as large structures through median region, decreasing gradually in size in posterior chaetigers, absent from last 10 or so chaetigers. Notopodial preacicular lobes rounded, rudimentary, neuropodial preacicular lobes of median chaetigers with interramal (dorsal) outgrowth (arrow), not fused with postacicular lobes. Acicular lobes deeply bilobed in anteriormost chaetigers, only slightly bilobed in median region, distinctly conical in posterior chaetigers. Neuropodial postacicular lobes elongate with rounded tips in median parapodia, gradually decreasing after C50–C60; dorsal cirri elongate and subulate in median chaetigers. Pharynx with long mid-dorsal subterminal papilla, proximal region covered with minute rounded papillae. Subtropical-boreal, shelf to slope and upper bathyal.		
–	(Key figure 27) Branchiae from C4–C6; neuropodial postacicular lobes of median chaetigers subequal in length to notopodial postacicular lobes or only slightly longer	**14**
14(13)	Branchiae from C4 (rarely from C5); in median chaetigers notopodial postacicular lobes obliquely oval, neuropodial postacicular lobes distinctly triangular with pointed (in juveniles) or rounded tips	***N. caeca***
(Key figure 28) Body length up to 250 mm, up to 150 chaetigers (Rainer 1991). Parapodial preacicular lobes poorly developed, rounded. Acicular lobe bilobed in anteriormost and median regions of large worms. Postacicular lobes extending well beyond acicular lobes; neuropodial postacicular lobes subequal in length to notopodial postacicular lobes or only slightly longer. Mid-dorsal subterminal papilla of pharynx similar in size to largest subterminal papillae or absent; proximal region covered with flattened warts. Arcto-boreal, upper shelf.		
–	Branchiae from C6; both noto- and neuropodial postacicular lobes wide, rounded	***N. assignis***
(Key figure 29) Body length up to 200 mm, up to 145 chaetigers (Hilbig 1997). Branchiae at first, minute, short and thick, gradually increasing in size through C12–C20, in median chaetigers large, recurved and cirriform, posteriorly decreasing in size, absent from last few chaetigers. Parapodial preacicular lobes poorly developed, rounded. Notopodial acicular lobes of median chaetigers deeply incised, neuropodial acicular lobes obliquely rounded. Postacicular lobes of median chaetigers foliaceous, extending well beyond acicular lobes; neuropodial postacicular lobes slightly longer than notopodial postacicular lobes. Pharynx without mid-dorsal subterminal papilla, proximally smooth. Tropical-boreal, sublittoral to shelf.		

**Key figure 5.**
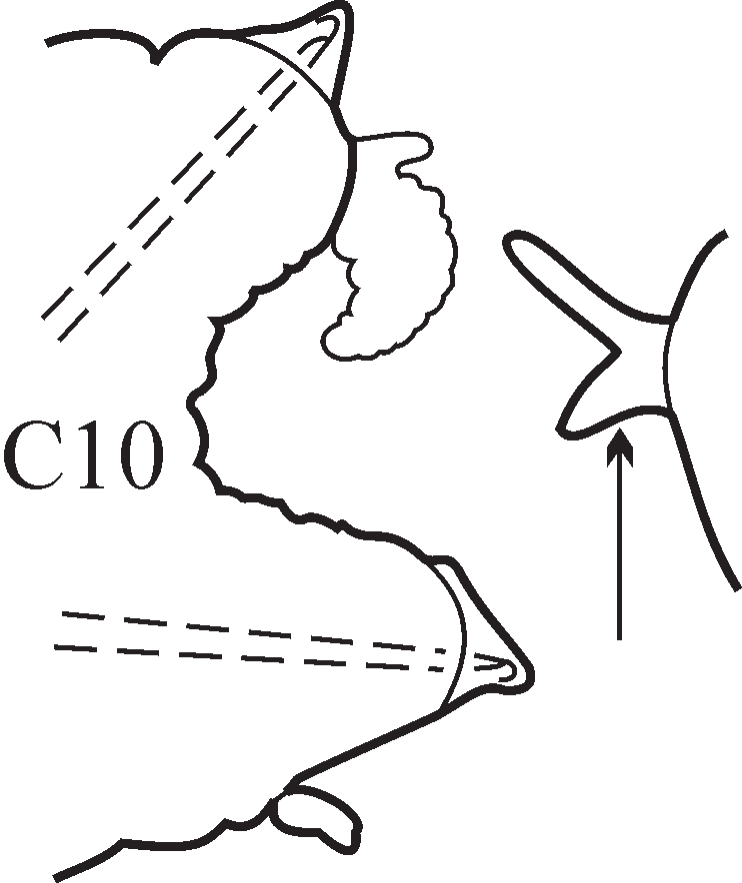


**Key figure 6.**
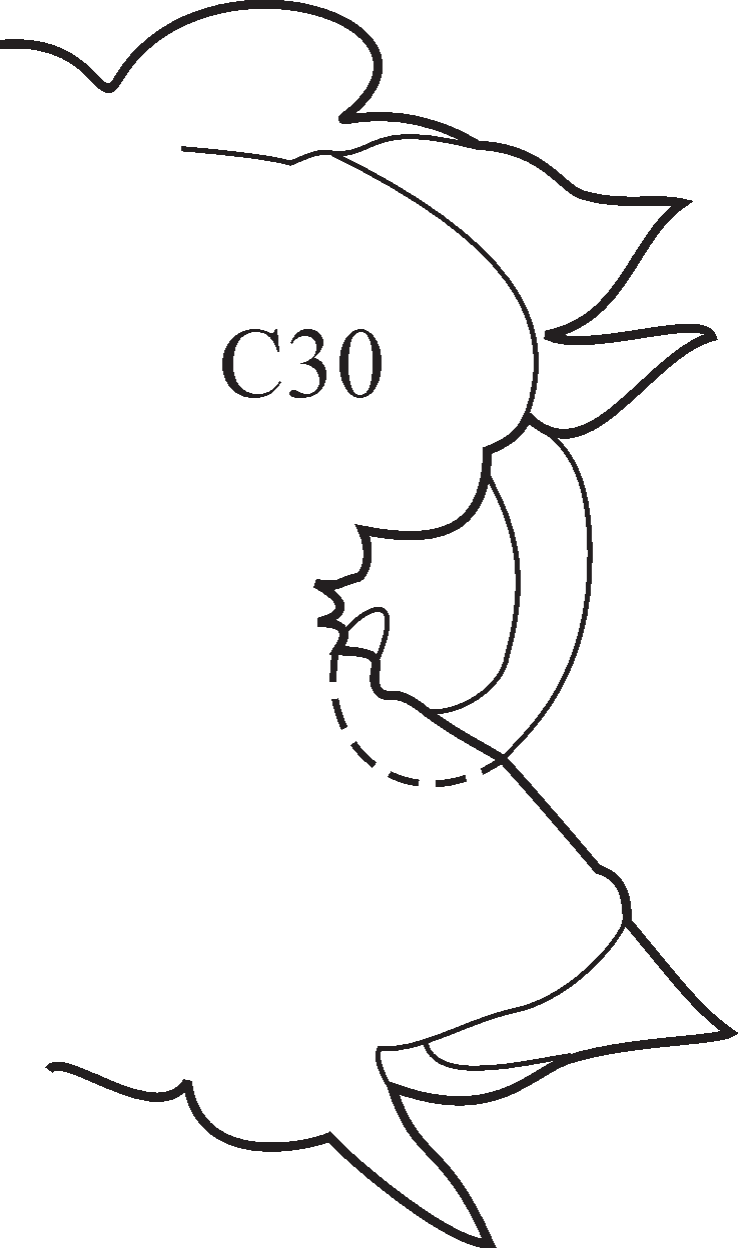


**Key figure 7.**
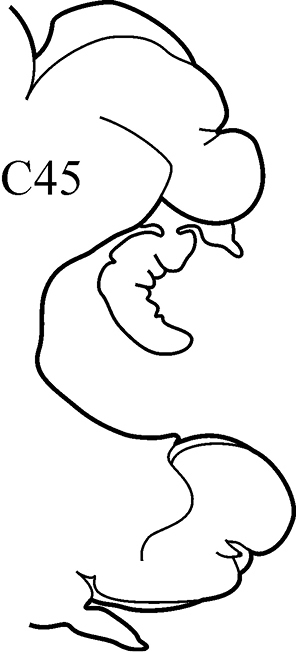


**Key figure 8.**
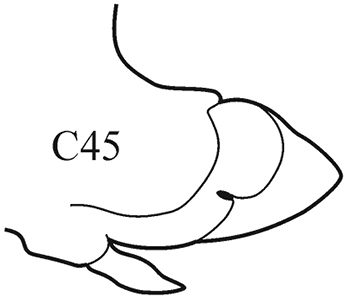


**Key figure 9.**
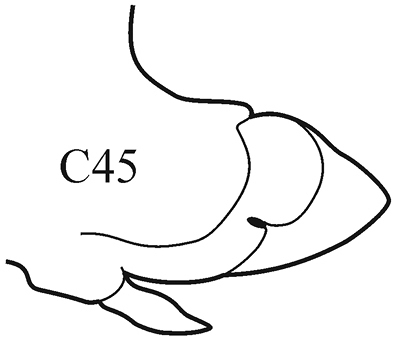


**Key figure 10.**
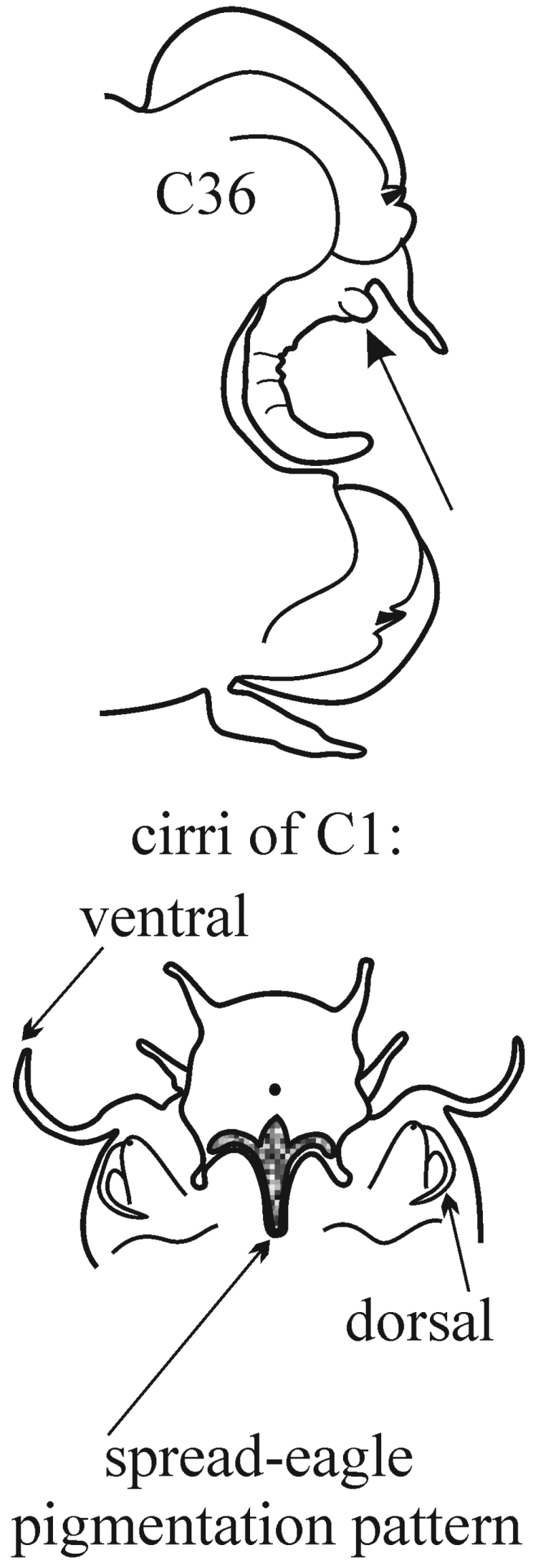


**Key figure 11.**
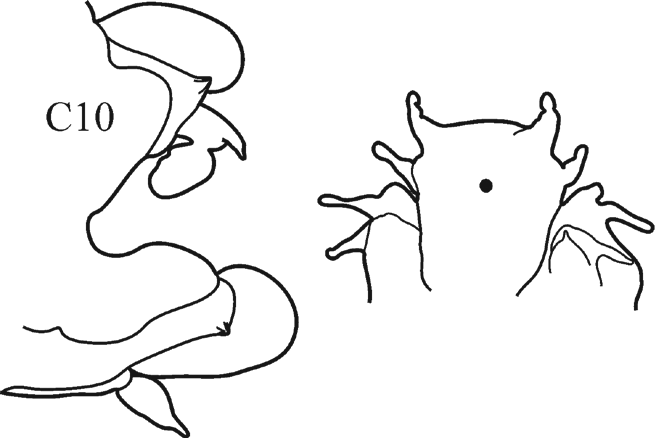


**Key figure 12.**
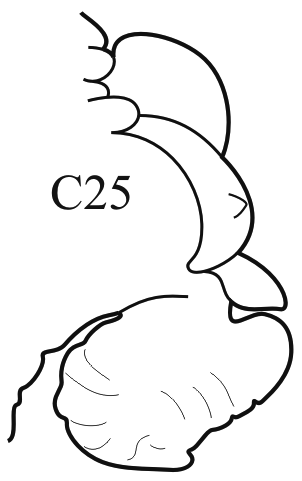


**Key figure 13.**
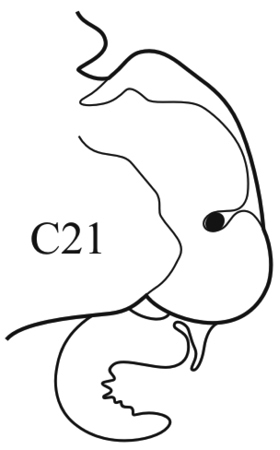


**Key figure 14.**
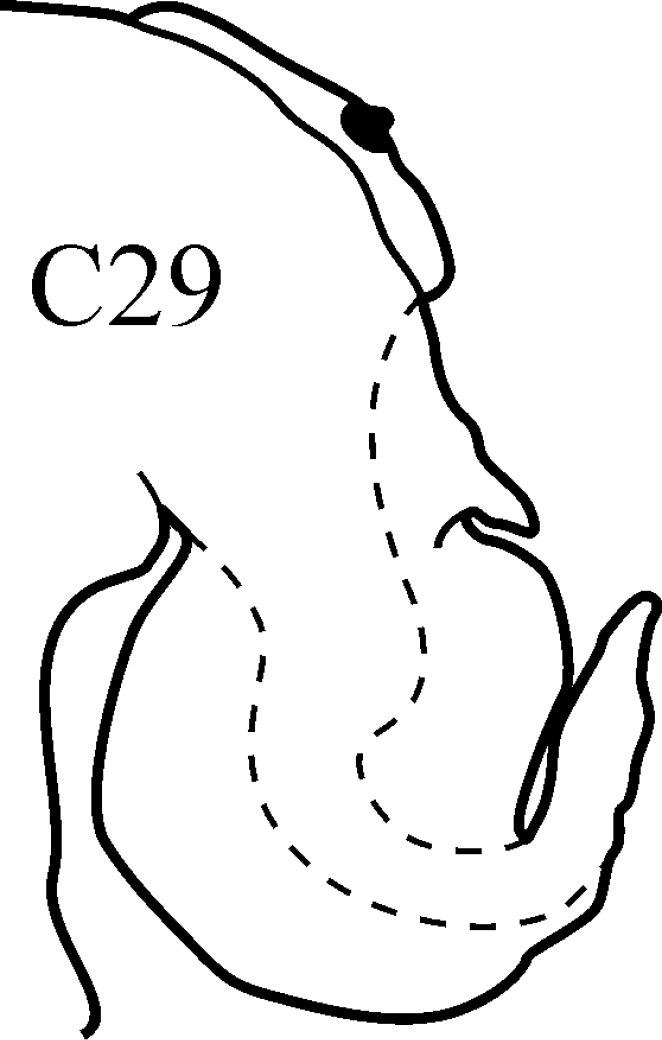


**Key figure 15.**
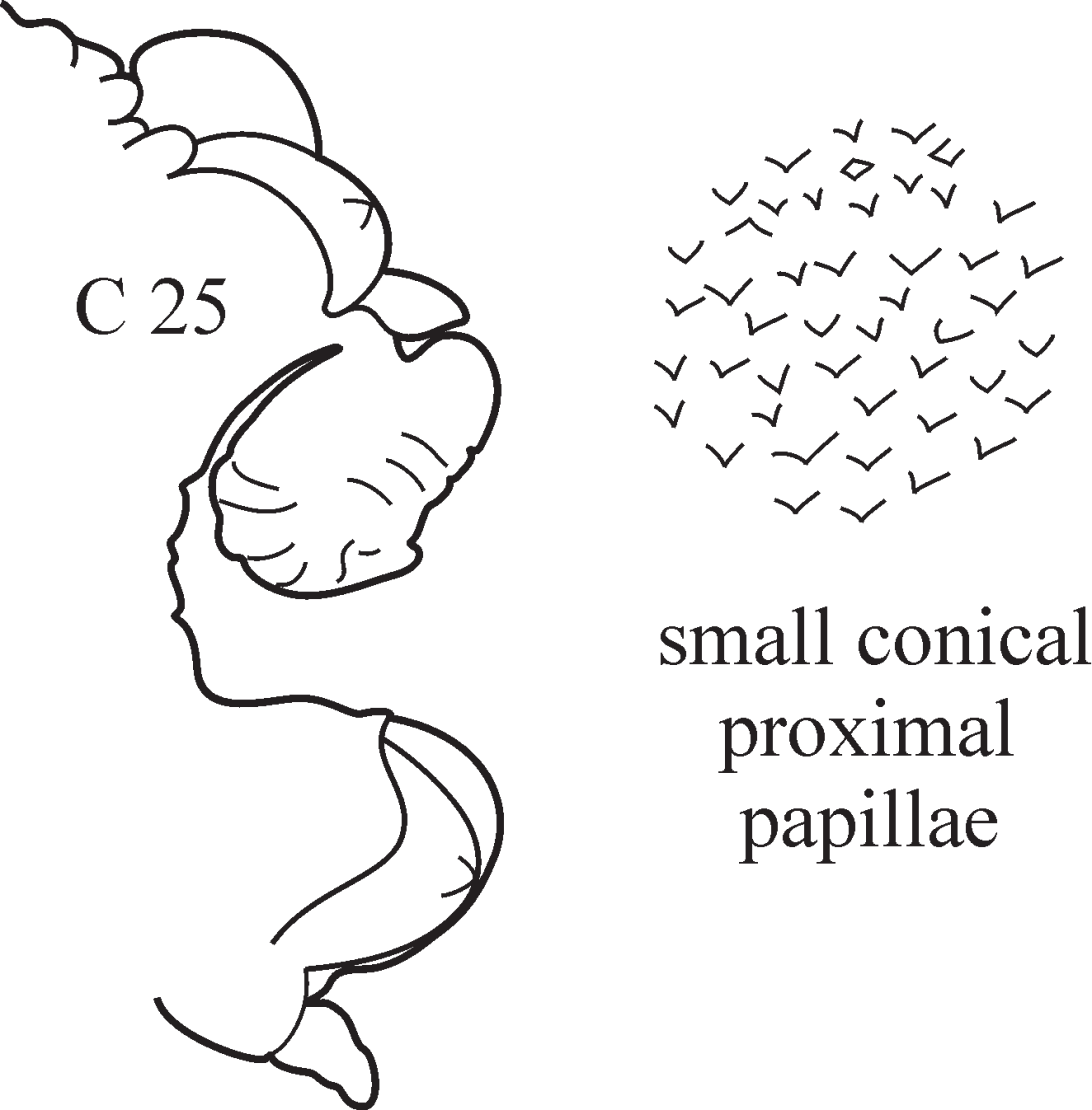


**Key figure 16.**
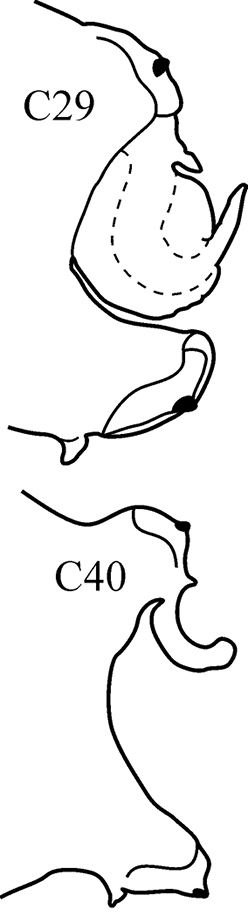


**Key figure 17.**
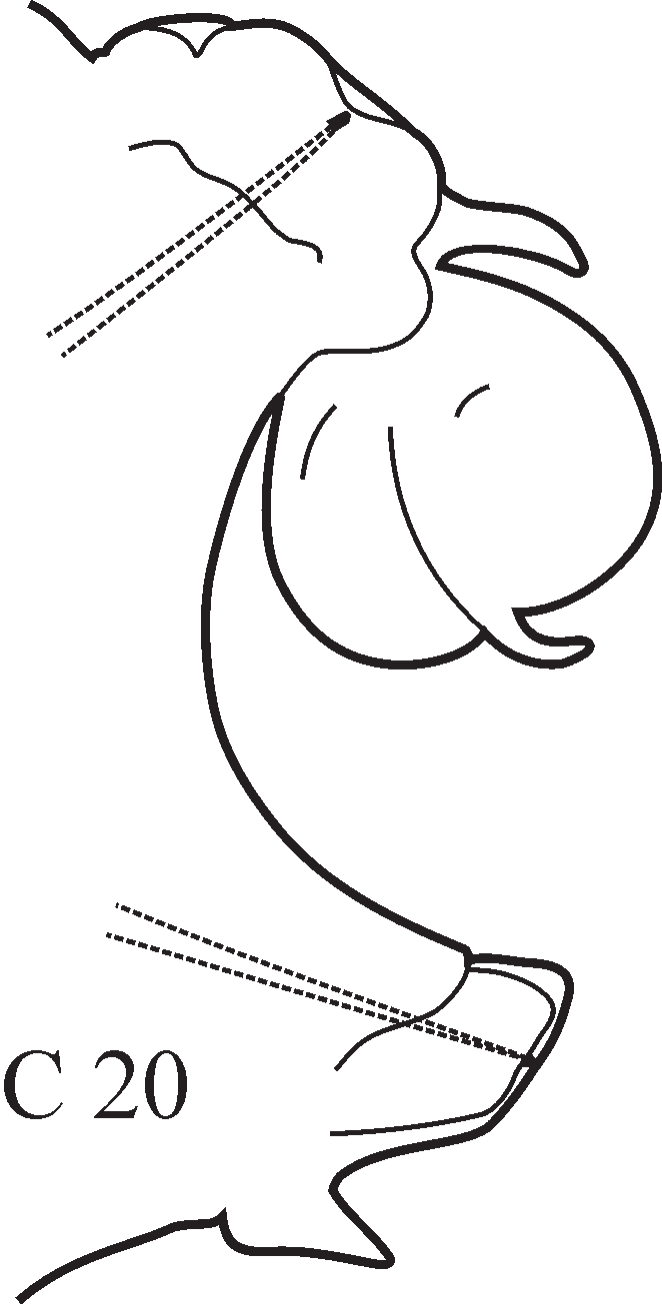


**Key figure 18.**
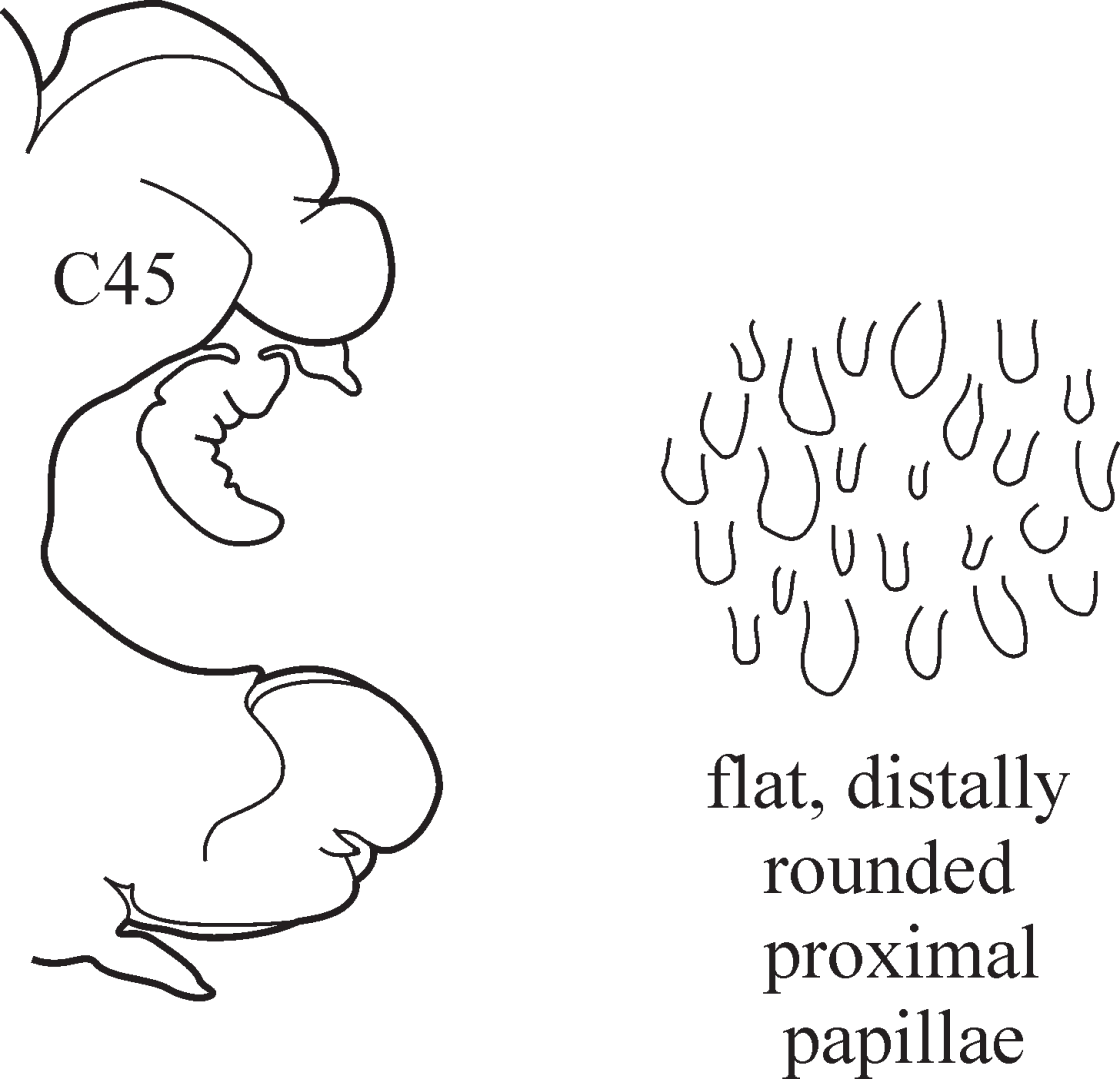


**Key figure 19.**
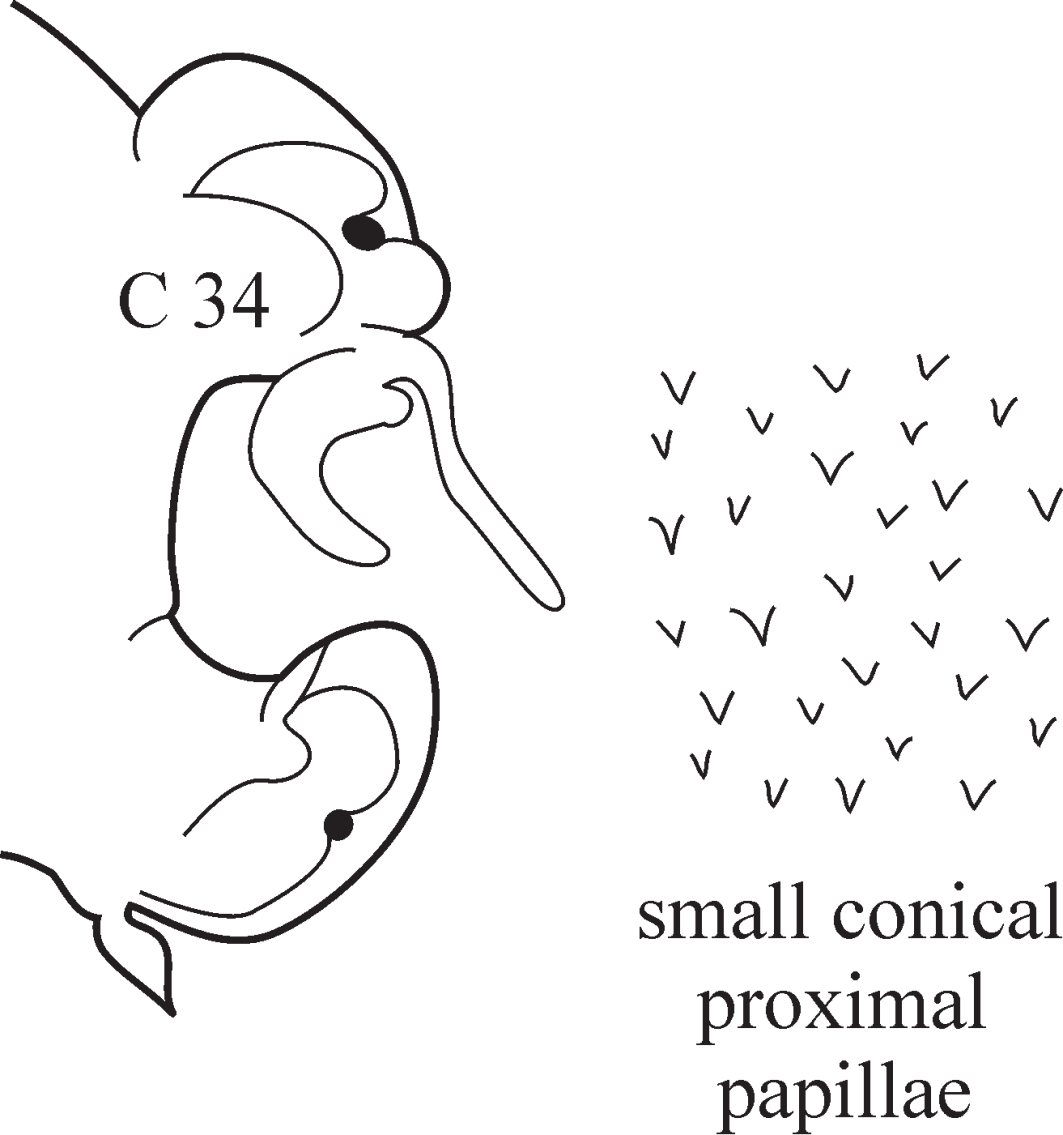


**Key figure 20.**
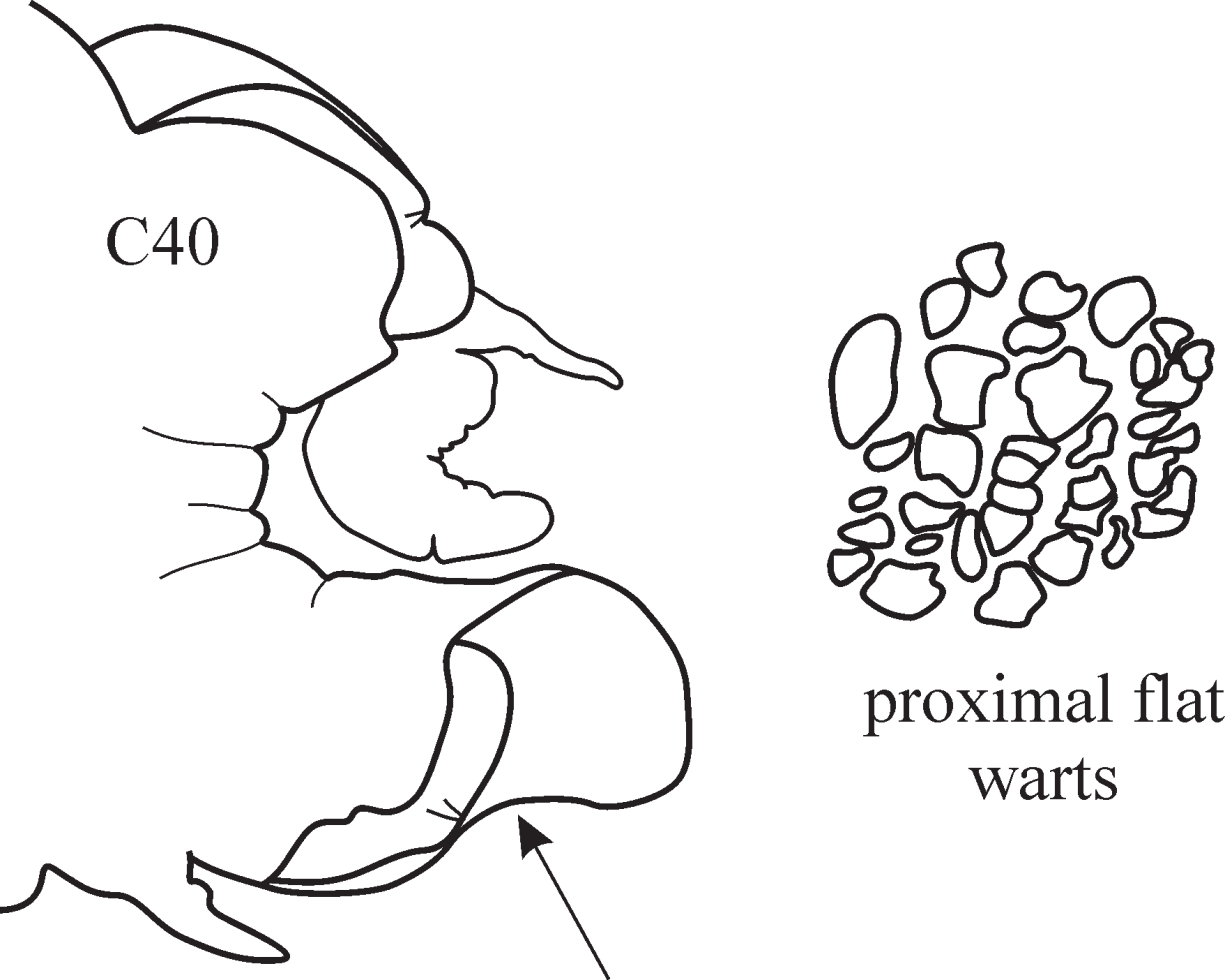


**Key figure 21.**
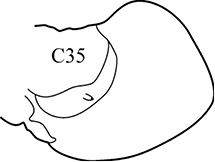


**Key figure 22.**
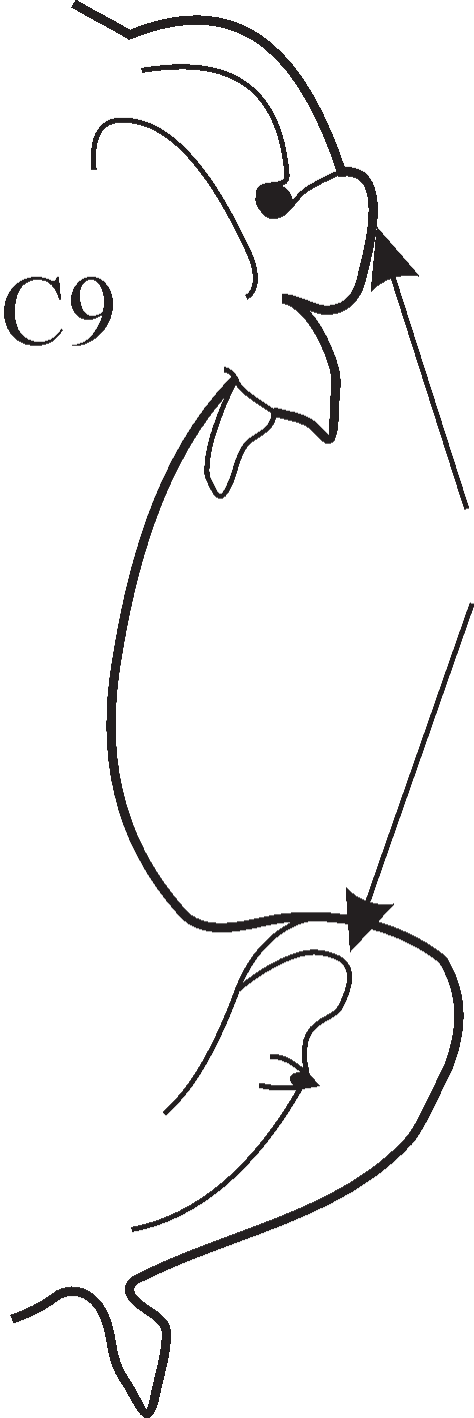


**Key figure 23.**
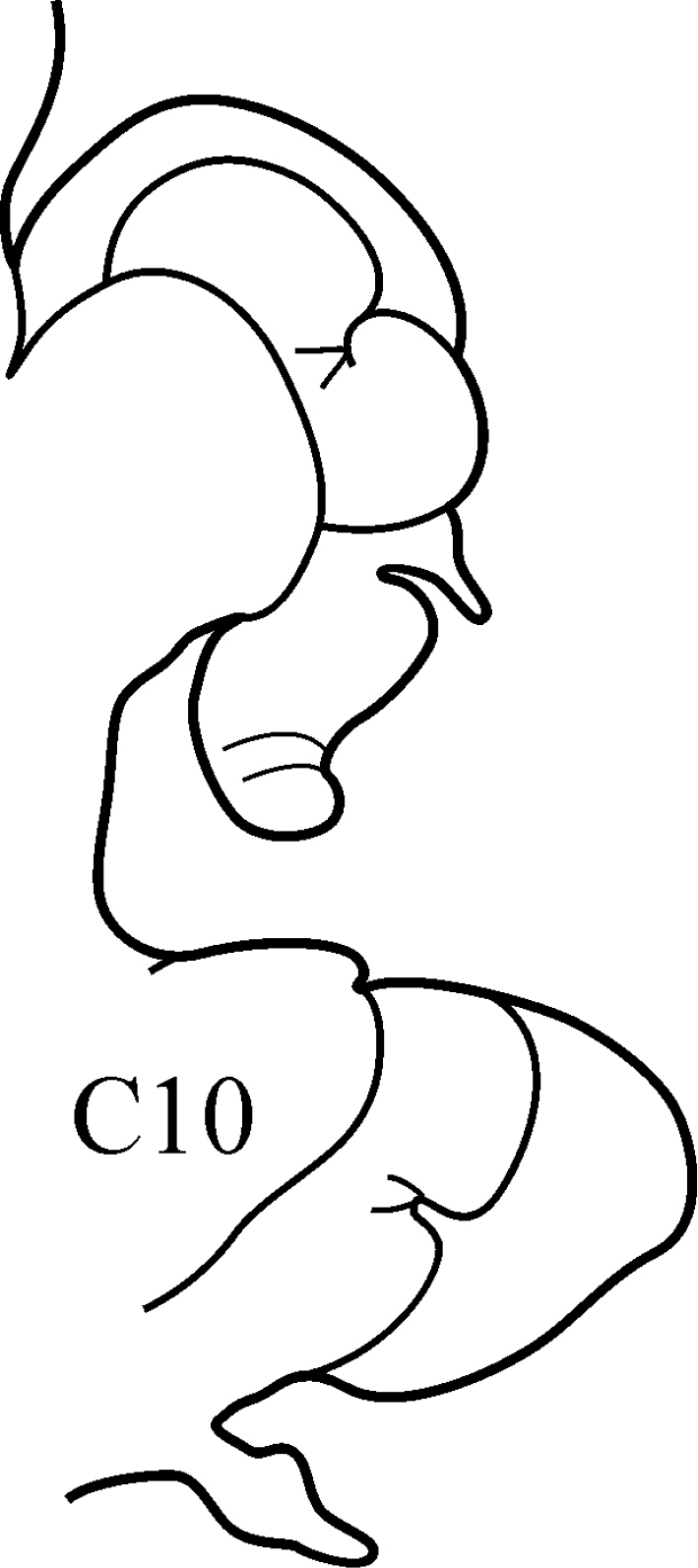


**Key figure 24.**
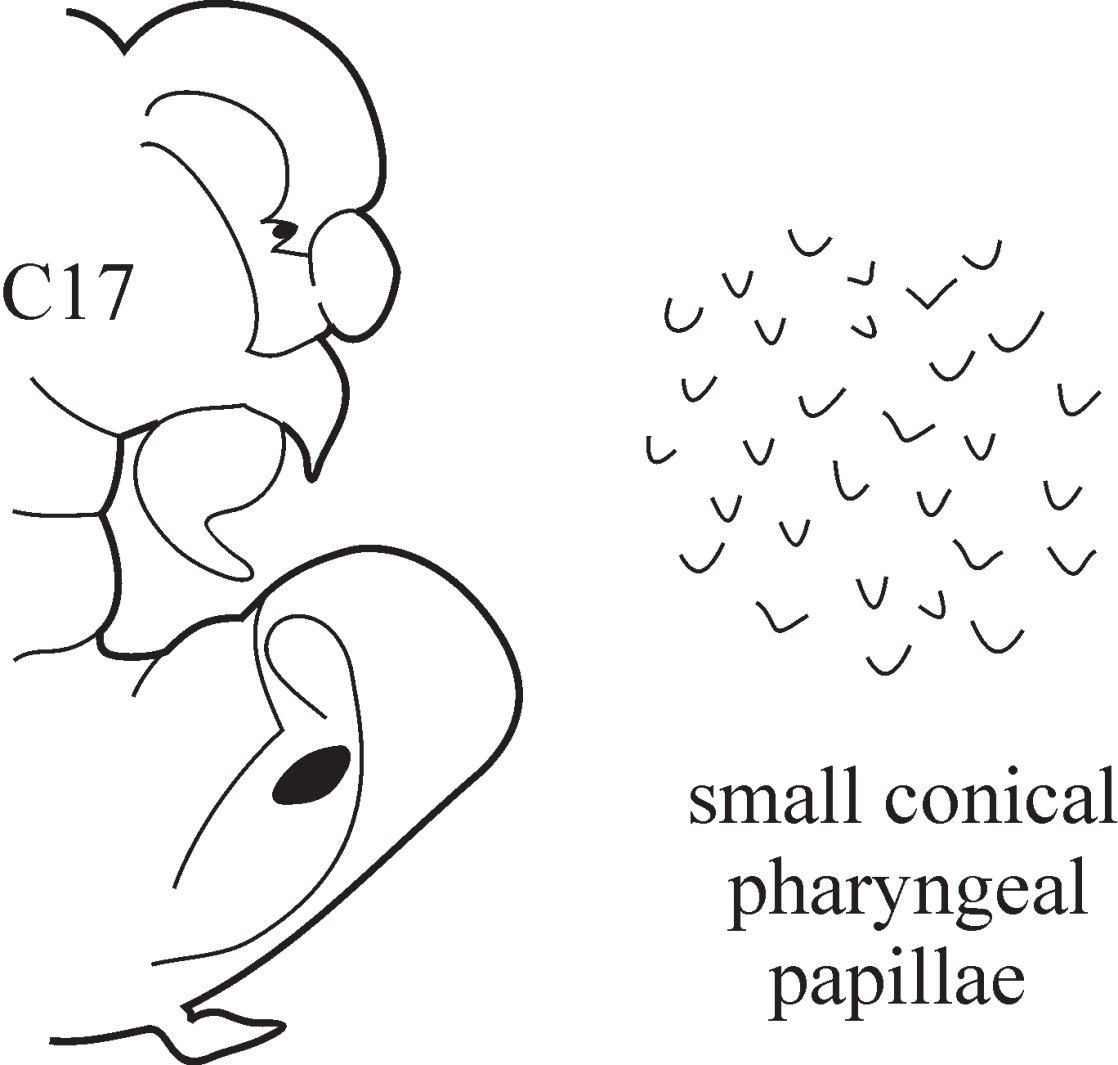


**Key figure 25.**
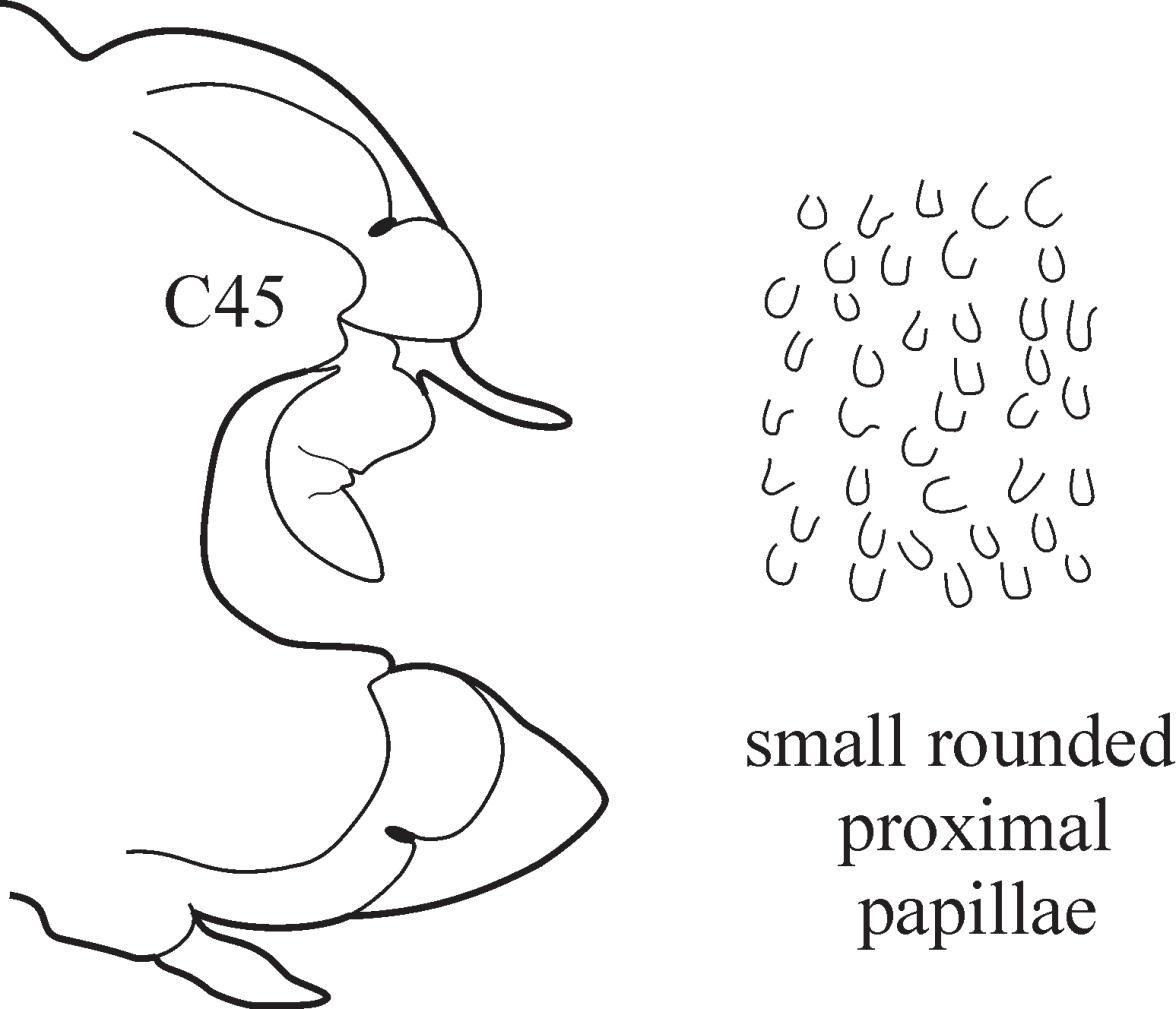


**Key figure 26.**
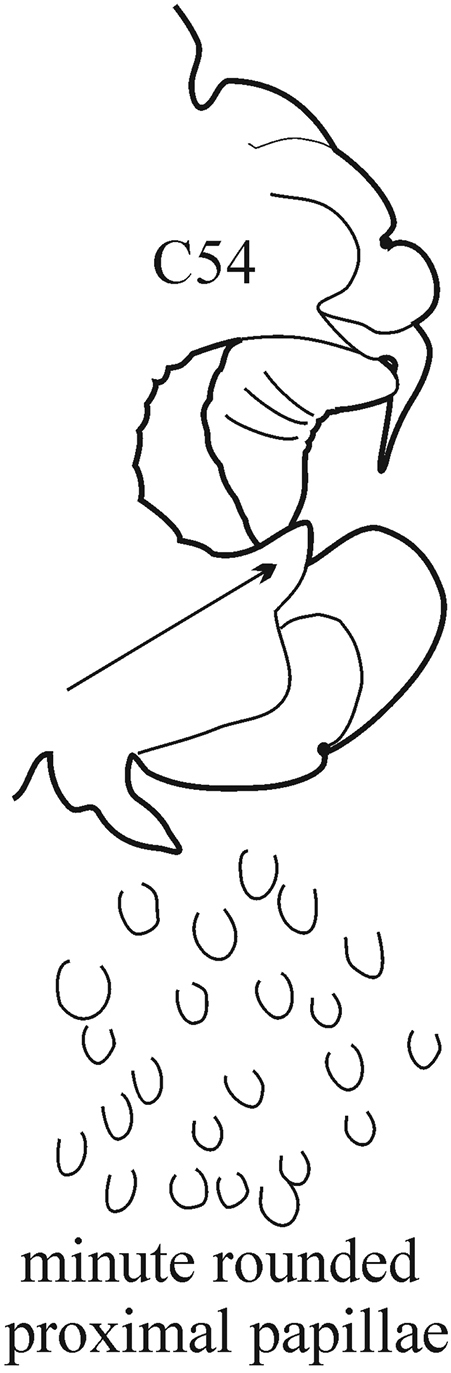


**Key figure 27.**
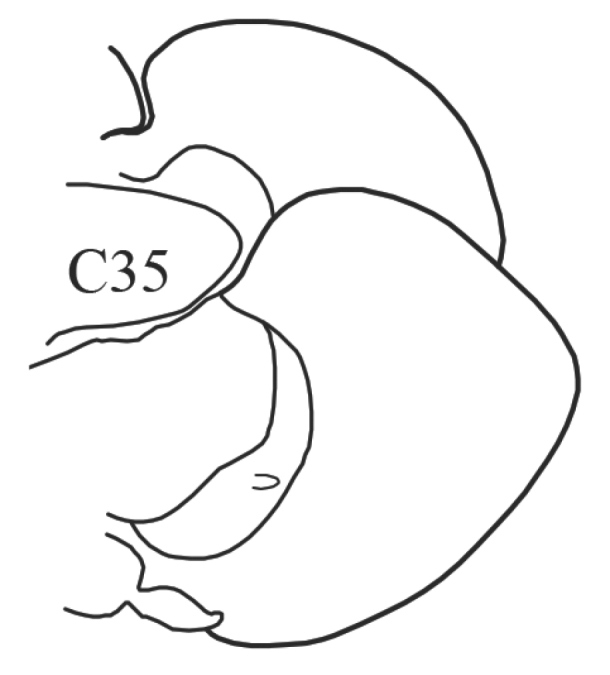


**Key figure 28.**
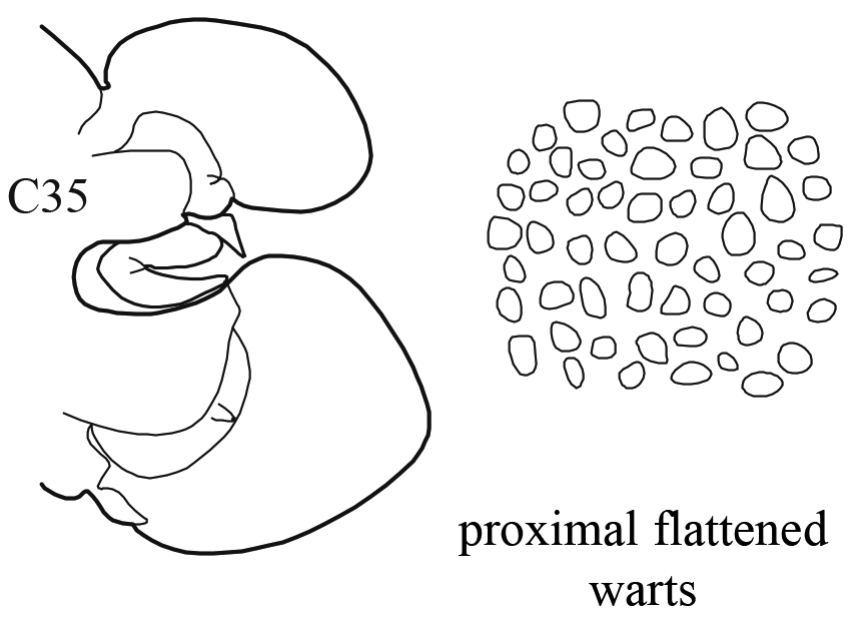


**Key figure 29.**
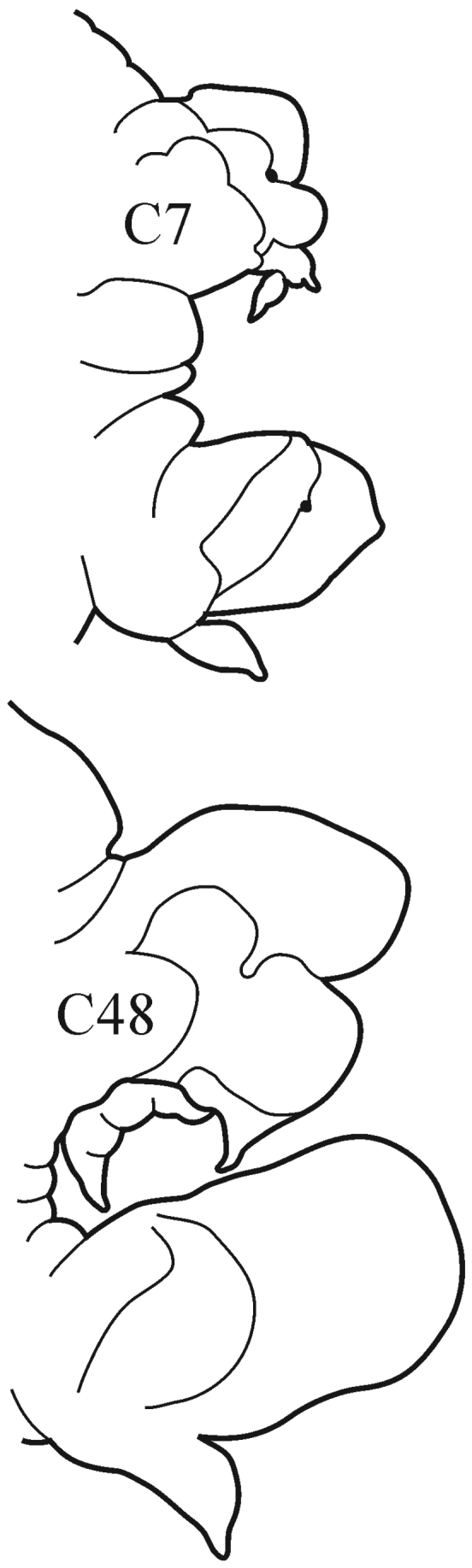


## List of Nephtyidae inhabiting the Sea of Okhotsk

***Aglaophamus*** Kinberg, 1865

*Aglaophamus malmgreni* (Théel, 1879)

***Micronephthys*** Friedrich, 1939

*Micronephthys minuta* (Théel, 1879)

***Nephtys*** Cuvier, 1817

*Nephtys assignis* Hartman, 1950

*Nephtys brachycephala* Moore, 1903

*Nephtys caeca* (Fabricius, 1780)

*Nephtys californiensis* Hartman, 1938

*Nephtys ciliata* (Müller, 1789)

*Nephtys longosetosa* Örsted, 1842

*Nephtys neopolybranchia* Imajima & Takeda, 1987

*Nephtys paradoxa* Malm, 1874

*Nephtys pente* Rainer, 1984

*Nephtys punctata* Hartman, 1938

*Nephtys rickettsi* Hartman, 1938

*Nephtys sachalinensis* Alalykina & Dnestrovskaya, 2015

*Nephtys schmitti* Hartman, 1938
